# Exploring the perspectives and preferences for HTA across German healthcare stakeholders using a multi-criteria assessment of a pulmonary heart sensor as a case study

**DOI:** 10.1186/s12961-015-0011-1

**Published:** 2015-04-28

**Authors:** Philip Wahlster, Mireille Goetghebeur, Sandra Schaller, Christine Kriza, Peter Kolominsky-Rabas, on behalf of the National Leading-Edge Cluster Medical Technologies ‘Medical Valley EMN’

**Affiliations:** Interdisciplinary Centre for Health Technology Assessment (HTA) and Public Health (IZPH), Friedrich‐Alexander‐University Erlangen‐Nürnberg (FAU), National Cluster of Excellence, Medical Technologies – Medical Valley EMN, Schwabachanlage 6, 91054 Erlangen, Bavaria Germany; School of Public Health, Universiy of Montreal & LASER Analytica, 1405 TransCanada Highway, Suite 310, Montréal, QC H9P 2 V9 Canada

**Keywords:** Decision-making, Health technology assessment, Heart failure, Multi-criteria decision analysis, Stakeholder participation, Stakeholder perspective

## Abstract

**Background:**

Health technology assessment and healthcare decision-making are based on multiple criteria and evidence, and heterogeneous opinions of participating stakeholders. Multi-criteria decision analysis (MCDA) offers a potential framework to systematize this process and take different perspectives into account. The objectives of this study were to explore perspectives and preferences across German stakeholders when appraising healthcare interventions, using multi-criteria assessment of a heart pulmonary sensor as a case study.

**Methods:**

An online survey of 100 German healthcare stakeholders was conducted using a comprehensive MCDA framework (EVIDEM V2.2). Participants were asked to provide i) relative weights for each criterion of the framework; ii) performance scores for a health pulmonary sensor, based on available data synthesized for each criterion; and iii) qualitative feedback on the consideration of contextual criteria. Normalized weights and scores were combined using a linear model to calculate a value estimate across different stakeholders. Differences across types of stakeholders were explored.

**Results:**

The survey was completed by 54 participants. The most important criteria were efficacy, patient reported outcomes, disease severity, safety, and quality of evidence (relative weight >0.075 each). Compared to all participants, policymakers gave more weight to budget impact and quality of evidence. The quantitative appraisal of a pulmonary heart sensor revealed differences in scoring performance of this intervention at the criteria level between stakeholder groups. The highest value estimate of the sensor reached 0.68 (on a scale of 0 to 1, 1 representing maximum value) for industry representatives and the lowest value of 0.40 was reported for policymakers, compared to 0.48 for all participants. Participants indicated that most qualitative criteria should be considered and their impact on the quantitative appraisal was captured transparently.

**Conclusions:**

The study identified important variations in perspectives across German stakeholders when appraising a healthcare intervention and revealed that MCDA can demonstrate the value of a specified technology for all participating stakeholders. Better understanding of these differences at the criteria level, in particular between policymakers and industry representatives, is important to focus innovation aligned with patient health and healthcare system values and constraints.

**Electronic supplementary material:**

The online version of this article (doi:10.1186/s12961-015-0011-1) contains supplementary material, which is available to authorized users.

## Background

Health technology assessment (HTA) is defined by the European Network for HTA as a “*multidisciplinary process that summarizes information about the medical, social, economic and ethical issues related to the use of a health technology in a systematic, transparent, unbiased, robust manner. Its aim is to inform the formulation of safe, effective, health policies that are patient focused and seek to achieve best value*” [1]. Accordingly, HTA is an essential tool for health policy decision-making as it assesses the available evidence about new health technologies.

HTA and policy decisions are usually complex due to the multiple aspects considered and the extensive amount of evidence. Frequent gaps in the evidence and associated uncertainty also contribute to the challenges faced by decision makers. Complex interventions complicate this problem further. The implementation aspects of complex health interventions are an essential link to the desired health outcomes [2,3]. In turn, the success or failure in improving health outcomes is not always attributed to the complex intervention itself but to context- and implementation-related issues. In order to achieve a comprehensive assessment of complex technologies, a variety of different issues have to be assessed such as effectiveness, ethical, context, and implementation issues [2]. However, the different aspects of HTA are not systematically taken into account for health policymaking. The results are mainly presented side-by-side and decision-makers are struggling to evaluate contradicting outcomes of complex HTA (e.g., better medical outcome but worse social outcome) [4].

In addition, the current approaches in HTA and healthcare decision-making have some limitations regarding the integration of the diversity of stakeholders’ preferences and perspectives in their processes. On the one hand, patient and public involvement is gaining more and more attention from health policymakers [5]. On the other hand, health economic tools are not able to identify and address the multiple voices of healthcare stakeholders [6]. Daniels’ ethical framework of ‘accountability for reasonableness’ provides the foundation for fair evaluation of healthcare interventions and fair decision-making [7]. According to this framework, all reasons and criteria for funding healthcare have to be accessible to all stakeholders. The reasons must be based on principles that ‘fair-minded’ people would agree upon. The criteria should reflect a society’s value [8]. These issues are all of a fundamental democratic nature and thus constitute the basis for acceptability of decisions. An optimal scenario would be to have a societal consensus on a collective solution for society and all important stakeholders to address rationing issues and the decision-making process associated with these. This could be achieved by engaging all stakeholders and ensure consideration of all stakeholders perspectives, preferences, and constraints.

Comprehensive multi-criteria decision analysis (MCDA) provides a tool in this direction. Its methodological basis enables the exploration of stakeholders’ preferences and perspectives and to explicitly structure the broad range of criteria on which real life evaluations and decisions are based [9]. MCDA provides insights into the rationale behind decision-making processes [10]. The MCDA process is democratic by nature and consists of several steps. Firstly, the decision problem needs to be defined and structured, i.e., the identification of valuable healthcare interventions from a holistic perspective. Secondly, a set of mutually independent criteria is defined and weighted based on their importance to individual stakeholders involved in the process. Thirdly, the appraised interventions are assigned scores based on their performance for each criterion; this is performed based on data available, hence the importance of aligning data development with decision criteria. Finally, a value estimate is calculated by combining weights and scores. A number of MCDA methods are available [11], with various degrees of complexity, including direct methods, such as 5- or 10-point weighting scales (Kepner Tregoe [12]), ranking, point allocation, analytic hierarchy process (AHP) [13], or indirect methods such discrete choice experiments (DCEs) [14-17]. DCEs have been successfully employed when the number of outcomes is small, while AHP is cognitively demanding for participants. The hierarchical structure of AHP in addition to the high number of evaluated alternatives can appear too complex for participants [18]. In DCE studies, the number of criteria levels is an important issue. Scoring of criteria with two levels is mostly not sufficient to illustrate the real world. However, the addition of criteria levels would have increased the complexity of discrete choices for respondents [14,17,19,20]. For this study, we selected an existing, open source, comprehensive MCDA framework, developed collaboratively through input of various stakeholders and which meets the methodological requirements of completeness, redundancy, and mutual independence [21-23]. This pragmatic framework, tested, adapted, and used by several HTA agencies [24-26], provides several weight elicitation methods (www.evidem.org), and includes a set of relevant criteria to explore stakeholders perspectives and preferences regarding evaluation and decision making for healthcare interventions. The framework consists of a core quantitative MCDA model and a qualitative contextual tool, with a comprehensive range of criteria and sub-criteria, which allows for adaptation to context.

The objectives of this study were to explore perspectives and preferences, in the German context and across different types of stakeholders, when appraising healthcare interventions using multi-criteria assessment of a heart pulmonary sensor as a case study.

## Methodology

### Study design

The EVIDEM (EVIdence based Decision-Making) framework, designed to assess interventions in healthcare using quantitative and qualitative decision criteria, was selected. This established framework proposes a comprehensive range of criteria fulfilling the methodological requirements of MCDA models. Additionally, we provided synthesized data on the heart sensor for each of these criteria to make the online survey feasible and to allow exploration of perspectives from a broad range of stakeholders. The framework, which includes normative (i.e., what we should do?) and feasibility (what can be done?) criteria, was adapted for this study in the German context including its translation. The EVIDEM core model used in this study consisted of 14 universal, quantitative criteria, while the contextual tool included 8 qualitative criteria. A definition of all criteria and scales used in the survey is provided in the Additional file 1: Table S1. Stakeholders across the German healthcare continuum (developers, health policymakers, healthcare professionals, citizens, and researchers) were invited to participate in an online survey about the assessment of a pulmonary heart sensor.

In the first part of the survey, individual perspectives on what matters most in HTA, i.e., which criteria contribute the most to the value of healthcare interventions, was captured by weight elicitation independently of the intervention. Participants were asked to provide relative weights for each criterion of the MCDA core model from their individual perspective, but in the context of coverage decision for healthcare interventions in general. For this survey, we selected a 5-point weighting scale. Participants provided a relative weight for each decision criterion of the quantitative core model, using a 5-point scale (1 = lowest relative importance, 5 = highest relative importance). They were also asked whether the contextual criteria should be considered for coverage decisions (Additional file 1: Table S1). For qualitative criteria of the contextual tool, participants indicated whether each criterion should never, rarely, sometimes, often, or always be included in decision-making processes. Detailed information with a definition of each criterion was provided to participants (Additional file 1: Table S1).

In the second part of the survey, in order to explore perspectives and differences on how an intervention is evaluated with regard to its performance for each criterion, participants were asked to appraise a pulmonary heart sensor using an MCDA evidence matrix. The evaluation matrix included the following information for each criterion of the framework: i) available scientific and colloquial data obtained from a literature review supplemented by data analyses and ii) quantitative performance scores of this intervention for each criterion of the core model and qualitative impacts for each contextual criterion.

Participants scored the performance of the pulmonary sensor using a scoring scale with defined anchors for each criterion ranging from 0 to 3, except for the intervention outcomes criteria (I1 ‘Effectiveness’ , I2 ‘Tolerability and Safety, I3 ‘Patient-reported outcomes’) which also had a negative scale (−3 to +3) to capture worse outcomes. An additional box for every criterion was available for participants to indicate if the data was not sufficient to understand the performance of the sensor, which corresponded to a zero score (e.g., no value for the sensor derived from this criterion). For qualitative contextual criteria, participants indicated whether consideration of a given criterion had a positive, neutral, or negative influence on the decision about the sensor. The estimated value of the heart sensor was elicited by the performance scores.

The online survey was tested in advance by six participants to ensure optimal responses and understanding of the process and of the criteria to be considered.

### MCDA evidence matrix for the heart sensor

CardioMEMS, the assessed pulmonary heart sensor, enables the permanent surveillance of patients with chronic heart failure (New York Heart Association III), based on telemonitoring, using a microelectromechanical system. This intervention was selected as a relevant case study given the current developments and the need to guide future research and development in the field of cardiology and telemedicine [27]. The implementation of this device comes along with a change in healthcare processes and thus affects all involved stakeholders including patients and physicians.

The MCDA evidence matrix was populated with available data for each criterion identified through an extensive literature review supplemented by additional analyses due to the scarcity of data. The clinical efficacy for the sensor was identified by searching healthcare databases, including PubMed, ScienceDirect (EMBASE), and Scopus, as well as websites of HTA and regulatory agencies. Since the device is relatively new, available data was limited. The study team drafted the relevant HTA report, which served as background for the MCDA questionnaire. Clinical data was obtained from one high-quality randomized controlled trial [28] and a Food and Drug Administration report about the trial [29]. One randomized clinical trial of an implantable right ventricular pressure monitoring system [30] and four observational studies of implantable systems [31-34] were also identified. The studies’ outcomes indicated reduced hospital admissions for heart failure in connection with the use of the pulmonary heart sensor. However, these trials assess different device systems [31,33,34]. It can be deduced that medical devices from the same group can radically change the outcome. Data about epidemiology of heart failure (incidence, mortality, etc.) in Germany was obtained from the Federal Statistical Office and several academic publications [35-39]. The current treatment standards for heart failure were obtained from national and international clinical guidelines [40-42]. Economic data on the device was estimated based on epidemiological data and treatment costs according to the Disease Related Groups of heart failure in Germany.

### Study participants

In total, 100 stakeholder representatives were contacted by email to participate in the survey (20 from each group) with an emailed invitation letter describing the project. The five key responder groups represented key healthcare stakeholders including health professionals, health policymakers, including from the Federal Joint Committee (GBA), Institute for Quality and Efficiency in Health Care, Statutory Health Insurance (GKV-Spitzenverband), industry, citizens, and researchers. Participants were identified through personal and business networks of the research group and were asked to take their own perspective into account when providing the relative importance of decision criteria and appraising the selected intervention for reimbursement decisions.

### Data analyses

Weights, scores, and impact obtained from the participations were analyzed in Excel. Descriptive statistics were applied and mean and standard deviations (SD) calculated in Excel. Normalized weights and scores were combined for each criterion (thus representing the contribution of each criterion to the value estimate) and summed using a linear additive model to calculate the MCDA value estimates for each participant, for each group of stakeholders, and for all participants [23].

Descriptive statistics (mean scores, SDs) are reported for those criteria for which largest differences across groups were observed. The cut-off value for reporting differences between mean for stakeholders and the mean for the whole population were set at 0.008 for normalized weights (distributed to sum up to 1) and 0.2 difference for scores (on a scale of 0 to 1).

## Results

### Participants

In total, 54 participants completed the survey (54% response rate) as illustrated in Table 1. From these participants, 70% (38 participants) completed the second part of the survey regarding appraisal of the healthcare intervention. The surveyed population included stakeholders across the healthcare sector, comprised of by health professionals (13%), policymakers (16.7%), industry representatives (18.5%), citizens (20.4%), and healthcare researchers (31.5%).

**Table 1:** **Participant characteristics**

**Type of stakeholder**	**Number of respondents (n)**
Health professionals	7
Health policymakers	9
Industry	10
Citizens	11
Researchers	17
**Total**	54

### Perspectives and preferences of stakeholders on decision criteria

**Figure 1:**
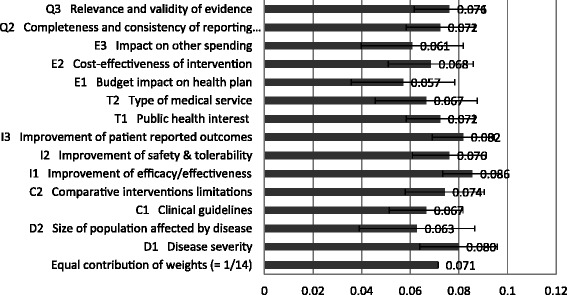
Relative weights for each criteria of the MCDA Core Model for all study participants. A 5-point weight elicitation technique was used (1 = low importance; 5 = high importance). The standard deviation of individual values and weights were normalized to sum up to 1.

Regarding relative weights provided by survey participants, Figure 1 shows that the most important criteria (normalized across criteria to sum up to 1) were ‘Improvements of efficacy/effectiveness’ (mean relative weight 0.086 [SD, 0.0125]), ‘Improvements in patient reported outcomes’ (0.082 [SD, 0.013]), ‘Disease severity’ (0.080 [SD, 0.016]), ‘Improvement in safety and tolerability’ (0.076 [SD, 0.015]), and ‘Relevance and validity of evidence’ (0.076 [SD, 0.014]). Least important criteria were ‘Budget impact on health plan’ (0.057 [SD, 0.021]), ‘Impact on other spending’ (0.061 [SD, 0.021]), and ‘Size of population’ (0.063 [SD, 0.024].

The largest variations in weights across participants were observed for ‘Size of population’ (SD, 0.024) and the criteria ‘Type of medical service’ (SD, 0.021), ‘Budget impact’ (SD, 0.021), and ‘Impact on other spending’ (SD, 0.021). The smallest variations were observed for ‘Improvements in effectiveness/efficiency’ (SD, 0.012) and the criteria ‘Public health interest’ , ‘Improvement in patient-reported outcomes’ , and ‘Completeness and consistency of the reported evidence’ (all SD, 0.013).

Regarding differences in weights between stakeholder groups, policymakers weighted the criteria ‘Budget impact’ and ‘Relevance and validity of evidence’ higher (+0.013 and +0.008, respectively) compared to all participants; they reported a lower relative weight for ‘Type of medical service’ (−0.011) and ‘Severity of disease’ (−0.008) (Additional file 2: Figure S1). From the perspective of health professionals, economic criteria were less important than for all participants, particularly ‘Budget impact’ (−0.018) and ‘Cost-effectiveness’ (−0.008); they also weighted the ‘Size of the affected population’ lower (−0.010) and the criterion ‘Limitations of comparable interventions’ higher (+0.014). Industry representatives put more weight on the ‘Cost-effectiveness of an intervention’ (+0.008) compared to all participants.

Regarding qualitative contextual criteria outlined in Table 2, a majority of respondents indicated that criteria ‘Goal of healthcare’ (92.45% responding often or always), ‘Fairness and priorities’ , ‘Opportunity costs and feasibility’ , ‘System capacity’ , and ‘Regulatory status’ should be considered often or always when making reimbursement decision on healthcare interventions. In contrast, a majority of respondents indicated that ‘political and historical context’ (35.84% for never or rarely) and ‘Pressures/barriers from stakeholders’ (60.38% for never or rarely) should never or rarely be considered in decision-making.

**Table 2:** **Participant responses to whether contextual criteria should be considered in coverage decisions on healthcare interventions (% of respondents)**

	**Never**	**Rarely**	**Sometimes**	**Often**	**Always**
**Et1 Utility – Goals of healthcare system/plan**	0.0%	0.0%	7.6%	37.7%	54.7%
**Et2 Fairness – Priorities and access of healthcare system**	1.9%	1.9%	15.1%	50.9%	30.2%
**Et3 Opportunity costs and affordability**	0.0%	3.8%	15.1%	56.6%	24.5%
**O1 System capacity and requirements**	3.8%	5.7%	30.2%	43.4%	17%
**O2 Political and historical context**	15.1%	20.8%	45.3%	15.1%	3.8%
**O3 Pressures/barriers from stakeholders**	26.4%	34%	32.1%	7.5%	0.0%
**O4 Environmental impact**	3.8%	17%	45.3%	17%	17%
**O5 Regulatory status**	3.8%	11.3%	22.6%	34%	28.3%

### Appraisal of the medical technology

**Figure 2:**
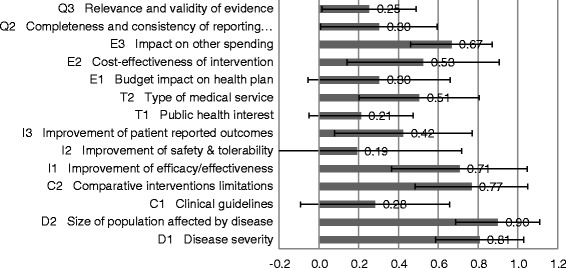
Performance scores of CardioMEMS for each criteria of the MCDA Core Model. Scoring scales of 0 to 3 were used for all criteria except −3 to +3 for clinical criteria (I1, I2, I3). These scores and the standard deviation of individual values were transformed to a scale of 0 to 1.

The quantitative appraisal revealed that the highest performance scores for the sensor outlined in Figure 2 were for the criteria ‘Size of the affected population’ (0.90 on a scale of 0 to 1 [SD, 0.3]), ‘Severity of disease’ (0.77 [SD, 0.3]), ‘Comparative intervention limitations’ (0.71 [SD, 0.4]), and ‘Improvement of efficacy/effectiveness’ (0.71 [SD, 0.34]). Lowest performance scores were observed for ‘Improvement of safety and tolerability’ (0.19 [SD, 0.53]), ‘Public health interest’ (0.21 [SD, 0.26]), and ‘Relevance and validity of evidence’ (0.25 [SD, 0.24]).

Consensus across participants on the performance scores for the sensor were most likely to be observed for ‘Size of population’ (SD, 0.2) and ‘Impact on other spending’ (SD, 0.2). The largest variations in scores across all stakeholders were observed for the criteria ‘Improvements in safety and tolerability’ (SD, 0.5) and ‘Clinical guidelines’ , ‘Budget impact’ , and ‘Cost-effectiveness’ (all SD, 0.4).

**Figure 3:**
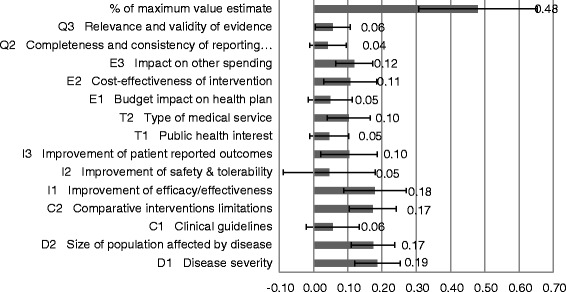
Value estimate of CardioMEMS and contribution of each criterion to the estimate. The standard deviation of individual values and the contribution of each criterion were normalized to sum up to 1.

These results were also reflected in the MCDA value estimate of 0.48 (on a scale of 0 to 1 or 48% of maximum value; Figure 3), obtained by combining normalized weights and performance scores. Major value contributors were the criteria ‘Severity of disease’ (0.19; SD, 0.07), ‘Size of affected population’ (0.17; SD, 0.06), ‘Comparative intervention limitations’ (0.17; SD, 0.07), and the ‘Improvement of efficacy/effectiveness’ (0.18; SD, 0.09).

Although statistical comparisons across stakeholder groups could not be performed due to a small sample size, overall differences were observed (Additional file 2: Figure S2B). Health professionals and academic researchers provided a lower score for the performance of the sensor on the criteria ‘Comparative intervention limitations’ (−0.2) compared to all participants. Health professionals scored ‘Improvement of efficacy/effectiveness’ for the heart sensor higher (+0.2) compared to all participants. The performance scores of the industry group were markedly higher for several criteria, particularly criteria of the clusters ‘Context of intervention’ (C1: +0.2, C2: +0.2), ‘Intervention outcomes’ (I1: +0.2, I2: +0.2, I3: +0.2), and ‘Budget impact’ (+0.4). Health policymakers provided lower scores compared to all participants, which was particularly marked for ‘Improvement of efficacy/effectiveness’ (−0.2) and ‘Cost-effectiveness’ (−0.3). On the other hand, the scores of the industry group were markedly higher for several criteria, particularly criteria of the clusters ‘Context of intervention’ (Disease severity: +0.2, Size of population: +0.2), ‘Intervention outcomes’ (Improvement of efficacy: +0.2, Improvement of safety: +0.2, Improvement of PRO: +0.2), and ‘Budget impact’ (+0.4). These variations resulted in the highest value estimate of the sensor of 0.68 (on a scale of 0 to 1, 1 representing maximum value) for the industry group and lowest estimate of 0.40 for policymakers, compared to 0.48 for all participants.

**Figure 4:**
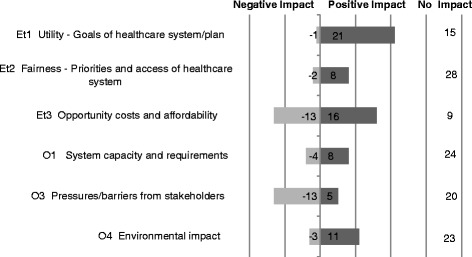
Impact of contextual criteria about CardioMEMS (sum of votes for positive (1), negative (−1), or neutral (0) impact).

Regarding qualitative contextual criteria, as illustrated in Figure 4, a majority of respondents agreed on the utility of the sensor (prevention of hospital stay) for the healthcare system, and thus its alignment with the goals of the healthcare system, as illustrated by a positive impact of consideration of the criterion on the quantitative value assessment. Consideration of environmental criteria had a positive impact on its value due to its degradability as it works without chemical battery. Consideration of pressures/barriers from stakeholders had an overall negative impact on the value of the sensor. Divergences were observed on the impact of considering opportunity costs and affordability on the overall value of the sensor.

## Discussion

This study identified issues about current perspectives on HTA in the German context as well as important variations across German stakeholders when appraising healthcare interventions. EVIDEM, as a decision support tool, was not directly designed to assess perspectives. Still, the perspectives of stakeholders translate into judgments at the criteria level. EVIDEM allowed exploration of these judgments with regards to two aspects: i) individual preferences and values on what matters most, or in other words, which criteria contribute the most to the worth of healthcare interventions in general (captured by weights independently of the intervention), and ii) understanding the value of a heart sensor measured by the contribution of a comprehensive range of criteria (elicited by performance scores).

Firstly, values and preferences provided the base for statements about the relative importance of assessment criteria. Other MCDA studies used DCEs to elicit preferences [14,15,17,20]. This is an issue which is also currently considered by the MCDA in Health Care Decision Making Emerging Good Practices Task Force of the ISPOR society. They recommend stated preference methods that “*are used to weight decision criteria*” [43]. Consequently, criteria weights can be regarded as preferences.

Secondly, stakeholders need to perceive and understand the complex evidence about health technologies, i.e., CardioMEMS. However, the perception and understanding of the provided evidence can be very different depending on the professional background of each stakeholder. As highlighted in our study, stakeholders scored the evidence very differently, depending on their personal background. Finally, the qualitative feedback contains both aspects: preferences and values as well as perception and understanding of the evidence.

Better understanding of these differences across stakeholders at the criteria level is important to focus innovation aligned with patient health and healthcare system values and constraints [44].

### Perspectives and preferences of stakeholders on decision criteria

Participants representing all types of stakeholders across the healthcare decision continuum in Germany indicated that the most important criteria for reimbursement decision making were ‘Improvement of efficacy/effectiveness’ , ‘Improvements in patient reported outcomes’ , ‘Disease severity’ , ‘Improvement in safety’ , and ‘Quality of evidence’ (relative weight >0.075 each). These results are to some extent in agreement with results of an international survey of decision criteria which indicated that most important decision criteria included ‘Clinical efficacy’ , ‘Safety’ , ‘Quality of evidence’ , ‘Disease severity’ , and ‘Impact on healthcare costs’ [45], a major difference being the importance of cost consideration.

This survey reveals the primary importance to German stakeholders related to the three criteria of the cluster ‘Intervention outcomes’ , including ‘Improvement in efficacy/effectiveness’ , ‘Improvement in safety’ , and ‘Improvement in patient reported outcomes’ , as well as the consensus on this point, revealed by the lowest SD across participants on their relative importance. This is in agreement with the current HTA approach in place in Germany through the GBA, which gives a high importance to ‘Incremental efficacy’ , ‘Incremental safety’ , and ‘Incremental patient reported outcomes’. Indeed, patient relevant outcomes (mortality, morbidity, and health-related quality of life) are the only criteria used in the assessment of the benefit of medical interventions [46].

However, the criterion ‘Disease severity’ was ranked third in this survey, revealing the importance of one of the fundamental objectives of healthcare to alleviate suffering in those who are worst off. However, current health policy does not actively take severity of disease into account [47]. The level of incremental benefit of a given healthcare intervention (defined as substantial, appreciable, moderate, present but not quantifiable, no benefit, or negative benefit) is determined by the GBA through a process that mentions that disease severity is considered, but it is not clearly stated how this is done [48]. Although stakeholder groups were too small to perform comparative statistical tests, the criterion ‘Disease severity’ appeared to be less important to policymakers compared to all participants, possibly pointing to a discrepancy between policies and the ethical implications associated with the concept of severity of diseases.

For policymakers, the criterion ‘Quality of the evidence’ was more important than for other stakeholders, revealing the value of evidence based decision-making in the German context. The GBA emphasizes the quality of evidence which needs to clearly demonstrate any claimed benefit [48].

For all participants, weights for economic criteria were generally lower than for other clusters of criteria, and ‘Budget impact of the intervention’ had the lowest weight of all criteria. Still, the highest SD (0.024) across all criteria indicated a poor agreement on the importance of economic issues. Across groups of stakeholders, the lowest weights on economic criteria were observed from the responses of health professionals, which reveal their wish to help patients without focusing on economic constrains [49-51]. These results are in line with currently implemented decision-making processes in Germany and the values associated with the process [52]. The German decision-making body (the GBA) assesses the incremental benefit of a new intervention with respect to an appropriate comparator. After a positive assessment, the economic aspects are considered taking the clinical benefit of the new intervention into account.

Regarding the qualitative assessment, a majority of stakeholders reported that criteria alignment of interventions with the ‘Goal of healthcare’ , ‘Fairness and priorities’ , ‘Opportunity costs and feasibility’ , ‘System capacity and implementation’ , and ‘Regulatory status’ should be considered in reimbursement processes. These qualitative but sometimes critical elements of decisions need a more formal integration into existing processes. Regarding the criteria ‘Political and historical context’ and ‘Pressures/barriers from stakeholders’ , full awareness of these aspects may be critical to make provisions to ensure acceptability and implementation of decisions [53,54], although a majority of participants indicated that these should not play a role in reimbursement decision making.

### Appraisal of the medical technology

Appraisal of the pulmonary heart sensor revealed that the major contributors to the value of this innovation were ‘Size of population’ , ‘Severity of disease’ , and ‘Comparative intervention limitations’ , highlighting the health need for the management of heart failure. ‘Improvement in efficacy/effectiveness’ was also a major contributor but limited data (one randomized controlled trial of 6 months duration) [28] was criticized as not being sufficient for informed decision-making. The quantitative appraisal of the pulmonary heart sensor revealed large differences in performance scores across participants for many criteria, with the largest SD in scores observed for the criteria ‘Improvements in safety and tolerability’ (SD, 0.5), ‘Clinical guidelines’ , and ‘Cost-effectiveness’ (both SD, 0.4). These variations may stem from different perspectives across participants, but given data limitations, and the fact that a survey format does not allow for discussion, uncertainty and/or misinterpretation might also have contributed to these large variations. Such differences in scoring were not observed in other settings in which the EVIDEM framework was applied to assess interventions by standing committees, and during which discussion and group interpretation of data took place before or during scoring [24,25].

Although stakeholder groups were too small to perform comparative statistical tests, a quantitative appraisal of the pulmonary heart sensor revealed large differences in scores between stakeholder groups. Health professionals scored ‘Improvement of efficacy/effectiveness’ for the heart sensor higher (+0.2 compared to all participants) while health policymakers scored this criteria lower (−0.2 compared to all participants). This suggests a more stringent judgment on what constitutes an improvement from health policymakers. The scoring scales of the MCDA framework used in the survey capture the judgment made on evidence, which results from an objective interpretation of evidence and a more subjective definition of what constitutes a major, moderate, minor, absence of improvement, or worsening of efficacy/effectiveness. Despite the caveats discussed above, this difference appears to quantitatively confirm the different viewpoints on the value of new interventions between innovation-oriented manufacturers and policymakers and purchasers of innovation in Germany [55].

This value estimate of 48% is of interest as far as it represents the contribution of each criterion to value, but the absolute value has limited interest in the absence of a frame of reference. Comparisons to other MCDA studies are not appropriate due to different sets of decision criteria and the application of other technical approaches, e.g., AHP. The comparison to other EVIDEM studies concludes certain reliability across different cultural, societal, and economic settings. MCDA estimates were 41% for growth hormone for Turner syndrome patients [23], 44% for Tramadol for chronic non-cancer pain [25], and 42 to 64% for 10 medicine appraisals in Canada [22]. Additionally, there is a 46% for coverage of a screening test for cervical cancer in South Africa [24]. However, it is not appropriate to compare the final estimates for several reasons. The criteria received different weights and the healthcare system and the cultural perspective differed from the German setting. Importantly, to the best of our knowledge, this is the first MCDA study using the EVIDEM 2.2 tool. Within this tool, scores can also be negative, e.g., if the intervention is less effective than the comparator. This factor contributes to a lower estimate value. For interpretation of the calculated value estimates, a comparison needs to be undertaken with other interventions in a second survey. When several interventions are appraised with such approaches by the same committee, as is the case in the HTA agency for the Lombardy region, where more than 20 interventions have been assessed with the MCDA approach, with a range of value estimates ranging between 0.22 and 0.72 (Michele Tringali, personal communication), such approaches are useful to rank interventions and guide decision-making at the system level.

As recommended by Baltussen et al. [56], MCDA estimates should be used as a guide to decision-making, rather than as a formula. This study revealed positive and negative impacts of qualitative criteria on the overall MCDA estimate. For example, by prompting participants to consider the environmental criteria, some element of value for the sensor could be captured qualitatively due to its degradability as it works without chemical batteries. Without a holistic framework, such considerations are unlikely to be brought to the discussion in line with results of an international survey which demonstrated that 30% of decision-makers currently consider environmental consequences of healthcare interventions but 70% indicated that it should be considered systematically [45].

### Limitations of the study

Study results should be considered in light of their limitations. We selected the 5-point weight elicitation technique and used linear scoring scales to keep the survey simple and to shorten time commitment. Still, it would be of interest to explore other weighting techniques and different types of scales as a follow-up to this exploratory study. In particular, the fact that differences in criteria weights had only limited influence on the overall value estimates raises some questions. The discriminatory power of the 5-point scale might be limited regarding the large number of criteria. As this was the first application of the EVIDEM framework to explore differences in criteria weights across stakeholder groups, this limitation should be taken into account for further research. Preston et al. [57] highlighted that scales with more categories (i.e., 7, 9, or 10) are most appropriate for most survey projects.

Our sampling approach (identification of participants through personal contact) has to be taken into account carefully when interpreting the presented results. However, this systematic sampling approach ensured a high response rate of 54%. Comparisons across groups of stakeholders are exploratory given the small sample size and the stochastic nature of the data, but nonetheless revealed differences. The lack of appropriate evidence, for most criteria, and difficulties in understanding the complex information resulted in lower scores and higher SDs, reflecting uncertainty on data and limitations in appraisal. Lay participants felt overtaxed by scientific evidence which is also true for patient representatives in other studies [17]. For stakeholders not working directly in a scientific field related to healthcare, graphical presentation and the teaching of basic statistical approaches can support the understanding of the provided information [58,59]. Despite the committee setting, these limitations can be partially overcome by discussion and input from experts for each criteria in order to facilitate interpretation of data; data limitations are a common issue in appraisals of health technologies [60].

## Conclusions

This study provided important insights into the current decision-making landscape in Germany and revealed that MCDA can demonstrate the value of a specified technology for all participating stakeholders. The application of a multi-criteria framework allowed to identify perspectives across German stakeholders when appraising a healthcare intervention at the criteria level, both quantitatively and qualitatively. A better understanding of these differences at the criteria level, in particular between policymakers and industry representatives, is important to focus innovations aligned with healthcare system values and constraints. The appraisal of the pulmonary heart sensor highlighted the health need for the management of heart failure. Multi-criteria frameworks provide a basis of dialogue between all stakeholders, which is beneficial for all parties [61], and which allows the definition of the most valuable interventions for patient and population health that can also contribute to sustainable, efficient, and equitable healthcare systems, thus facilitating access to patients [62,63]. By combining the benefits of both simple heuristic judgments (e.g., trade-offs) [64] and structured mathematical models [65], multi-criteria supports the complexity of evaluations and decision-making and provides a mean to elucidate and discuss variations in perspectives across stakeholders at the criteria level. Further research is needed to explore the role of multiple criteria to develop fair and accountable processes based on a better understanding of perspectives across the healthcare decision continuum within a society and across cultures. Such approaches can also provide some powerful analytical tools to identify how social values affect decision-making [66].

## Additional files

Additional file 1: Table S1. Definition of criteria included in the survey, quantitative scoring scales, and qualitative impacts for appraisal of the heart sensor. Additional file 2: Figure S1. (**A**) Subgroup analysis of differences in weights; only criteria which are different above the cut-off values between the mean and the subgroup mean are displayed (cut-off 0.008 for weight differences). (**B**) Subgroup analysis of differences in scores; only criteria which are different above the cut-off values between the mean and the subgroup mean are displayed (cut-off 0.2 for score differences), different MCDA value estimates per subgroup are reported.

## Abbreviations

AHP: Analytic Hierarchy Process
DCE: Discrete Choice Experiments
EVIDEM: Evidence Based Decision Making
GBA: Federal Joint Committee
HTA: Health technology assessment
MCDA: Multi-criteria decision analysis
