# Supplementary material for: Exploring the perspectives and preferences for HTA across German healthcare stakeholders using a multi-criteria assessment of a pulmonary heart sensor as a case study
Source: Health Res Policy Syst. 2015 Apr 28;13:24. doi: 10.1186/s12961-015-0011-1 (PMC4424515; doi:10.1186/s12961-015-0011-1)
Supplement: Additional file 1: Table S1. — Definition of criteria included in the survey, quantitative scoring scales, and qualitative impacts for appraisal of the heart sensor. [file 12961_2015_11_MOESM1_ESM.doc]

**Additional file 1: Table S1: Definition of criteria included in the survey, quantitative scoring scales and qualitative impacts for appraisal of the heart sensor**

| **Criterion** |  | |
| --- | --- | --- |
| **Criteria of the MCDA Core Model** | **Definition of criterion** | **Quantitative scoring scales**  **(low and high end of scales)** |
| **Disease impact** |  |  |
| D1 – Disease severity | Severity of the health condition of patients treated with the proposed intervention (or severity of the health condition that is to be prevented) with respect to mortality, disability, impact on quality of life, clinical course (i.e., acuteness, clinical stages). | Not severe (0),  Very severe (+3) |
| D2 - Size of population | Number of people affected by the condition (treated or prevented by the proposed intervention) among a specified population at a specified time; can be expressed as annual number of new cases (annual incidence) and/or proportion of the population affected at a certain point of time (prevalence). | Very rare disease (0)  Common disease (+3) |
| **Context of intervention** |  |  |
| C1 - Clinical guidelines | Concurrence of the proposed intervention (or similar alternatives) with the current consensus of experts on what constitutes state-of-the-art practices in the management of the targeted health condition; guidelines are usually developed via an explicit process and are intended to improve clinical practice. | No recommendation (0)  Strong recommendation (+3) |
| C2 - Comparative interventions limitations | Shortcomings of comparative interventions in their ability to prevent, cure, or ameliorate the condition targeted; also includes shortcomings with respect to safety, patient reported outcomes and convenience. | No or very minor limitations (0)  Major limitations (+3) |
| **Intervention outcomes** |  |  |
| I1 - Improvement of efficacy/ effectiveness | Capacity of the proposed intervention to produce a desired (beneficial) change in signs, symptoms or course of the targeted condition above and beyond beneficial changes produced by alternative interventions. Includes efficacy and effectiveness data, as available. | Lower efficacy/effectiveness than comparators presented(-3)  Major improvement in efficacy/ effectiveness (+3) |
| I2 - Improvement of safety & tolerability | Capacity of the proposed intervention to produce a reduction in intervention-related harmful or undesired health effects compared to alternative interventions. | Lower safety/tolerability than comparators presented (-3)  Major improvement in safety/ tolerability (+3) |
| I3 - Improvement of patient reported outcomes | Capacity of the proposed intervention to produce beneficial changes in patient-reported outcomes (PROs) (e.g., quality of life) above and beyond beneficial changes produced by alternative interventions; also includes improvement in convenience to patients. | Worse PRO (-3)  Major improvement (+3) |
| **Type of benefit** |  |  |
| T1 - Public health interest | Risk reduction provided by the proposed intervention at the population-level (e.g., prevention, reduction in disease transmission, reduction in the prevalence of risk factors). | No risk reduction (0)  Major risk reduction (+3) |
| T2 - Type of medical service | Nature of the clinical benefit provided by the proposed intervention at the patient-level (e.g., symptom relief, prolonging life, cure). | Minor service (0)  Major service (+3) |
| **Economics** |  |  |
| E1 - Budget impact on health plan | Net impact of covering the intervention on the budget of the target health plan (excluding other spending, see E3). This represents  the differential between expected expenditure for the proposed intervention and potential cost savings that may result from replacement of other intervention(s) currently covered by the health plan. Limited to cost of intervention (e.g. acquisition cost, implementation and maintenance cost). | S Substantial budget impact (0)  Substantial budget reduction (+3) |
| E2 - Cost-effectiveness of intervention | Ratio of the incremental cost of the proposed intervention to its incremental benefit compared to alternatives. Benefit can be expressed as number of events avoided, life-years gained, quality-adjusted life-years gained, additional pain-free days etc. | Not cost-effective (0)  Highly cost-effective (+3) |
| E3 - Impact on other spending | Impact of providing coverage for the proposed intervention on other expenditures (excluding intervention cost, see E1) such as hospitalization, specialist consultations, adverse events, long-term care, disability costs, lost productivity, caregiver time etc. | Substantial additional other spending (0)  Substantial reduced spending (+3) |
| **Quality of evidence** |  |  |
| Q2 - Completeness and consistency of reporting evidence | Extent to which reporting of evidence on the proposed intervention is complete (i.e., meeting scientific standards on reporting) and consistent with the sources cited. | Many gaps /inconsistent (0)  Complete and consistent (+3) |
| Q3 - Relevance and validity of evidence | Extent to which evidence on the proposed intervention is relevant to the decision-making body (in terms of population, disease stage, comparator interventions, outcomes etc.) and valid with respect to scientific standards (i.e., study design etc.) and conclusions (agreement of results between studies). This includes consideration of uncertainty (e.g., conflicting results across studies, limited number of studies & patients). | Low relevance /validity (0)  High relevance /validity (+3) |

| **Contextual Criteria** | **Defintion** | **Qualitative Impact on assessment** |
| --- | --- | --- |
| Et1 - Utility - goals of healthcare | Goal of healthcare is to maintain normal functioning. Such consideration is aligned with the principle of utility, which considers the act to produce the greatest good or “greatest benefits for the greatest number”. Mission and scope of a health plan/system derive from this principle. | Positive impact (+1)  Neutral impact (0)  Negative impact (-1) |
| **Et2 - Fairness –population priority & access** | Priorities for specific groups of patients are defined by societies/decisionmakers and reflect their moral values. Such considerations are aligned with the principle of fairness, which considers treating like cases alike and different cases differently and often gives priority to those who are worst-off (theory of justice). | Positive impact (+1)  Neutral impact (0)  Negative impact (-1) |
| **Et3 - Efficiency –opportunity costs & affordability** | Opportunity costs include resources or existing interventions that may be forgone if intervention under scrutiny is used/reimbursed. Such consideration is aligned with the principle of efficiency, which considers maximizing impact on health for a given level of resources (efficiency can be considered at the patient level and at societal level). This criterion also covers the concept of affordability. Both affordability and opportunity cost considerations require a financial/budgeting exercise. | Positive impact (+1)  Neutral impact (0)  Negative impact (-1) |
| **O1 - System capacity & appropriate use of intervention** | The capacity of healthcare system to implement the intervention and to ensure its appropriate use depends on its infrastructure, organization, skills, legislation, barriers and risks of inappropriate use. Such considerations include mapping current systems and estimating whether the use of the intervention under scrutiny requires additional capacities (note: if available, economic data on these aspects could be included under the economic criterion E3 of the MCDA model). | Positive impact (+1),  Neutral impact (0),  Negative impact (-1) |
| **O2 - Stakeholder pressures/barriers** | Pressures/barriers from groups of stakeholders or individuals are often part of the context surrounding healthcare interventions. Such considerations include being aware of pressures and interests at stake and how they may affect values of decisionmakers. | Positive impact (+1)  Neutral impact (0)  Negative impact (-1) |
| **O3 - Political/ historical context** | Political/historical context may influence the value of an intervention in consideration of specific political situations and overall priorities (e.g., priority to innovation) as well as habits, traditions and precedence. | Positive impact (+1)  Neutral impact (0)  Negative impact (-1) |
| **O4 - Environmental impact** | Extent to which the production and use or implementation of the intervention causes environmental damages | Positive impact (+1)  Neutral impact (0)  Negative impact (-1) |
| **O5 - Regulatory status** | Current regulatory status of an intervention and the adherence to the requirements of market approval | Positive impact (+1)  Neutral impact (0)  Negative impact (-1) |
