# Supplementary material for: Exploring the perspectives and preferences for HTA across German healthcare stakeholders using a multi-criteria assessment of a pulmonary heart sensor as a case study
Source: Health Res Policy Syst. 2015 Apr 28;13:24. doi: 10.1186/s12961-015-0011-1 (PMC4424515; doi:10.1186/s12961-015-0011-1)

**Additional file 2 Figure S1 A: Subgroups analysis of differences in weights; only criteria which are different above the cut-off values between the mean and the subgroup mean are displayed (cut-off 0.008 for weight differences)**


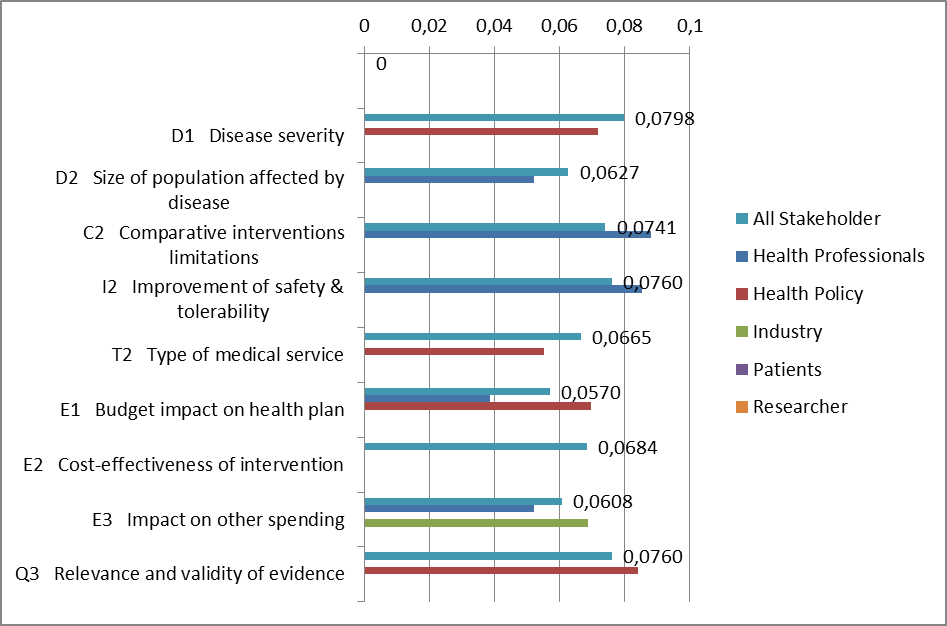


**Figure S1 B: Subgroups analysis of differences in scores; only criteria which are different above the cut-off values between the mean and the subgroup mean are displayed (cut-off 0.2 for score differences), different MCDA value estimates per subgroup are reported**


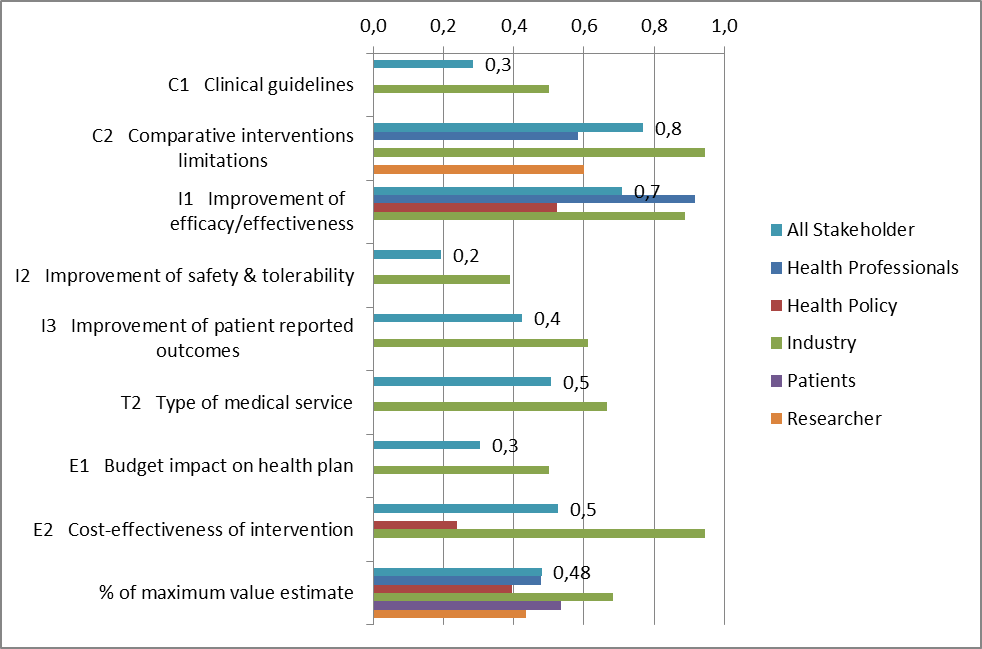

Supplement: Additional file 2: Figure S1. — (A) Subgroup analysis of differences in weights; only criteria which are different above the cut-off values between the mean and the subgroup mean are displayed (cut-off 0.008 for weight differences). (B) Subgroup analysis of differences in scores; only criteria which are different above the cut-off values between the mean and the subgroup mean are displayed (cut-off 0.2 for score differences), different MCDA value estimates per subgroup are reported. [file 12961_2015_11_MOESM2_ESM.doc]
